# Inflammatory and nutritional markers predict the risk of post-operative delirium in elderly patients following total hip arthroplasty

**DOI:** 10.3389/fnut.2023.1158851

**Published:** 2023-11-02

**Authors:** Wenhao Hu, Ziyi Song, Houlai Shang, Jingcheng Wang, Yuedong Hao

**Affiliations:** ^1^Department of Orthopedic Surgery, The Affiliated Huaian No.1 People’s Hospital of Nanjing Medical University, Huai’an, Jiangsu, China; ^2^Department of Orthopedics, Subei People’s Hospital of Jiangsu Province, Yangzhou, Jiangsu, China

**Keywords:** post-operative delirium, total hip arthroplasty, inflammatory markers, nutritional markers, nomogram

## Abstract

**Objectives:**

This study intended to explore whether albumin-associated inflammatory and nutritional markers could predict post-operative delirium (POD) in older patients after total hip arthroplasty (THA). In addition, we established a nomogram model for POD prediction.

**Methods:**

Totally, 254 elderly cases who received THA were included. Clinical and laboratory data of these patients were retrospectively collected. Albumin-associated inflammatory and nutritional markers included neutrophil-to-albumin ratio (NAR), CRP-to-albumin ratio (CAR), prognostic nutritional index (PNI), and systemic inflammation score (SIS). The LASSO, univariate and multivariate logistic regression analyses were utilized to screen risk factors. A nomogram model was developed according to the results of multivariate regression analyses.

**Results:**

Among 254 patients, 49 cases had POD with an incidence of 19.3%. LASSO regression and multivariate logistic analyses suggested that preoperative NAR, preoperative PNI, preoperative SIS, and age >75 years were risk factors for POD. A nomogram model was developed according to the results of multivariate logistic analyses. The calibration curve suggested that the predicted probability of this nomogram model was in good line with the actual probability. The DCA showed that this nomogram model had net benefits for the prediction of POD for elderly patients following THA.

**Conclusion:**

Albumin-associated inflammatory and nutritional markers including NAR, PNI, and SIS could predict POD in elderly patients following THA.

## 1. Introduction

Delirium, a neuropsychiatric syndrome, is characterized by an acute change in awareness, attention and cognition. It could affect up to 15–50% of elderly patients undergoing surgery (1). A meta-analysis showed that the overall incidence of delirium was 23% (2). Multiple risk factors included acute medical illness, drug use, trauma or surgery could trigger delirium (3). Delirium was reported to be associated with prolonged hospital stay time, higher complications, higher morbidity, and mortality (4–7). Although delirium was a common syndrome, most of cases were undocumented and unrecognized by the clinicians (8–10). Therefore, identifying biomarkers may help to the early detection of delirium, which is necessary for patients and clinicians.

Delirium is a primary post-operative complication in elderly patients with total hip arthroplasty (THA) (11, 12). A recent meta-analysis revealed that the incidences of post-operative delirium (POD) in patients with THA or total knee arthroplasty (TKA) varied significantly (0–48%), with a median incidence of 14.8%; most of the studies ranged from 10 to 15% (11). Although the pathogenesis of delirium is poorly understood, inflammation was a well-established factor involved in the development of delirium (13–15). In addition, studies suggested that hypoalbuminemia was an independent risk factor for POD in surgical patients (16–18), indicating that malnutrition was related to the risk of POD. Albumin (Alb), a protein in acute inflammatory response, is used to evaluate the nutritional status of patients receiving surgery. Therefore, we aimed to investigate whether Alb-derived markers integrating inflammation and nutrition could be the significant predictors for POD in elderly patients following THA. These Alb-associated inflammatory and nutritional markers were as follows: neutrophil-to-albumin ratio (NAR), CRP-to-albumin ratio (CAR), prognostic nutritional index (PNI), and systemic inflammation score (SIS).

## 2. Patients and methods

### 2.1. Participants

The enrollment flowchart of elderly patients receiving THA is presented in Supplementary Figure 1. Eventually, 254 patients following THA were included in this study from January 2019 to December 2022. Patients were included when they conformed to the following criteria: (1) patients received THA for the first time; (2) patients were Han nationality; and (3) age >60 years. Exclusion criteria included: (1) patients with neurological or psychiatric disorders; (2) patients with post-operative infections; (3) patients did not have complete data; (4) patients had a family history of mental illness; and (5) patients used antipsychotic medications in recent 3 months. All cases signed informed consent. This study was approved by the Ethics Committees of the Affiliated Huaian No.1 People’s Hospital of Nanjing Medical University. This study was in line with the *Helsinki declaration*.

### 2.2. Diagnosis of POD

The diagnosis of POD was based on the criteria of Confusion Assessment Method (CAM) (19). The CAM instrument had the following four criteria: (I) inattention, (II) acute onset and fluctuating course, (III) altered consciousness, and (IV) disorganized thinking. The diagnosis of delirium required the presence of criterion (I) and (II), and either criterion (III) or (IV). Within 7 days after surgery, POD status was assessed for patients receiving THA. POD was determined at the same period every-day. Patients would not be evaluated after surgery beyond 7 days.

### 2.3. Data collection

The baseline characteristics including age, smoking, sex, hypertension, drinking, body mass index (BMI), diabetes mellitus, position of THA, anesthesia time, and surgery time were collected. Routine laboratory indexes were performed, including neutrophil, monocyte, lymphocyte, CRP, and Alb. The blood indicators were collected after admission within 24 h. The definitions of lymphocyte/monocyte ratio (LMR), NAR, neutrophil/lymphocyte ratio (NLR), SIS, CAR, and PNI are shown in Supplementary Table 1.

### 2.4. Statistical analysis

The data were presented as numbers (%) or median (with interquartile range) or means (± standard deviations). Categorical variables were calculated by Fisher exact test or Chi-square test, while continuous variables were determined by *t*-test (normally distributed variables) or Mann-Whitney *U*-test (non-normally distributed variables). The variance inflation factor (VIF) and tolerance were used to evaluate collinearity between variables. LASSO regression, and univariate and multivariate logistic analyses were utilized to find independent risk factors. Receiver operating characteristic (ROC) curve analysis was performed to evaluate the predictive ability of markers for POD. The C-index value, calibration plots, and decision curve analysis (DCA) were analyzed in the nomogram model, which was based on the findings of multivariate logistic analyses. A *P*-value < 0.05 indicated statistically significant. SPSS (version 21.0, Chicago, IL, USA), MedCalc software, R software (version 4.1.3), and Graphpad Prism (version 8.0) were used.

## 3. Results

### 3.1. Patient characteristics

**FIGURE 1:**
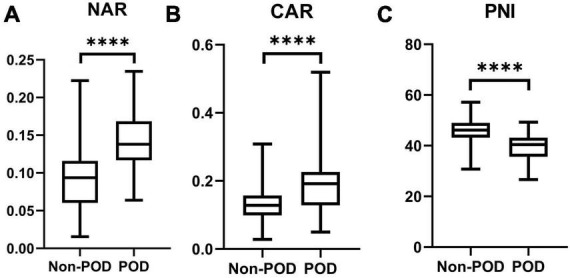
The preoperative values of NAR **(A)**, CAR **(B)**, and PNI **(C)** between POD group and non-POD group (****indicated *P* < 0.001).

A total of 254 elderly cases after THA were analyzed, including 167 females (65.7%) and 87 males (34.3%). The average age of cases was 68.07 ± 5.68 years. Forty-nine cases with POD occurred, with a proportion of 19.3% (Table 1). The age of POD group was older than that in non-POD group (Table 1). The THA position between these two groups was significantly different (Table 1). The anesthesia time of POD group was significantly longer than that in non-POD group (Table 1). No significant differences were shown in sex, BMI, smoking, drinking, hypertension, diabetes mellitus, position, and surgery time (Table 1). The results of laboratory tests for these patients are listed in Table 2. The cases with SIS = 0 or 1 or 2 were 33 (13.0%), 115 (45.3%), and 106 (41.7%), respectively. Cases were divided into low group (SIS = 0 or 1) and high group (SIS = 2). The values of NAR and CAR in POD group were remarkedly higher than that in non-POD group; the value of PNI in POD group was markedly lower than that in non-POD group (Figure 1).

**TABLE 1:** Clinicopathological characteristics for elderly patients following total hip arthroplasty.

Variables	Overall	Non-POD	POD	*P*-value
Sample size, *n*	254	205	49	
Sex, *n* (%)				0.351
Male	87 (34.3%)	73 (35.6%)	14 (28.6%)	
Female	167 (65.7%)	132 (64.4%)	35 (71.4%)	
Age, mean (SD), years	68.07 ± 5.68	67.25 ± 5.02	71.51 ± 6.93	<0.001*
BMI, mean (SD), kg/m^2^	24.84 ± 3.74	24.99 ± 3.83	24.21 ± 3.29	0.190
Smoking, *n* (%)				0.888
Yes	17 (6.7%)	13 (6.3%)	4 (8.2%)	
No	237 (93.3%)	192 (93.7%)	45 (91.8%)	
Drinking, *n* (%)				0.726
Yes	10 (3.9%)	9 (4.4%)	1 (2.0%)	
No	244 (96.1%)	196 (95.6%)	48 (98.0%)	
Hypertension, *n* (%)				0.898
Yes	81 (31.9%)	65 (31.7%)	16 (32.7%)	
No	173 (68.1%)	140 (68.3%)	33 (67.3%)	
Diabetes mellitus, *n* (%)				0.934
Yes	32 (12.6%)	26 (12.7%)	6 (12.2%)	
No	222 (87.4%)	179 (87.3%)	43 (87.8%)	
Position, *n* (%)				<0.001*
Left	132 (52.0%)	97 (47.3%)	35 (71.4%)	
Right	119 (46.9%)	107 (52.2%)	12 (24.5%)	
Double	3 (1.1%)	1 (0.5%)	2 (4.1%)	
Surgery time, mean ± SD, h	1.82 ± 0.55	1.80 ± 0.53	1.93 ± 0.62	0.135
Anesthesia time, mean ± SD, h	2.16 ± 0.60	2.12 ± 0.59	2.31 ± 0.65	0.045*

*P-value < 0.05. BMI, body mass index; POD, post-operative delirium.

**TABLE 2:** Laboratory tests of elderly patients following total hip arthroplasty.

Variables	Non-POD	POD
Neutrophil, median (IQR), 10^9^/L	3.73 (2.46–4.72)	4.95 (4.11–5.63)
Monocyte, median (IQR), 10^9^/L	0.45 (0.29–0.61)	0.53 (0.42–0.72)
Lymphocyte, median (IQR), 10^9^/L	1.07 (0.82–1.47)	0.86 (0.59–1.17)
Albumin, median (IQR), g/L	40.70 (37.95–43.10)	35.50 (32.00–38.40)
CRP, median (IQR), mg/L	5.20 (4.10–6.40)	6.50 (4.55–8.15)
NAR, median (IQR)	0.09 (0.06–0.12)	0.14 (0.12–0.17)
LMR, median (IQR)	2.33 (1.49–4.68)	1.62 (0.96–2.30)
CAR, median (IQR)	0.13 (0.10–0.16)	0.19 (0.13–0.23)
PNI, median (IQR)	46.15 (43.18–49.03)	40.40 (35.65–43.15)
**SIS**		
0	33 (16.1%)	0 (0%)
1	106 (51.8%)	9 (18.4%)
2	66 (32.2%)	40 (81.6%)

IQR, interquartile range; CRP, C-reactive protein; NAR, neutrophil/albumin ratio; LMR, lymphocyte/monocyte/ratio; CAR, CRP/albumin ratio, PNI, prognostic nutritional index; SIS, systemic inflammation score.

### 3.2. Diagnostic value of Alb-associated inflammatory and nutritional markers for POD

**FIGURE 2:**
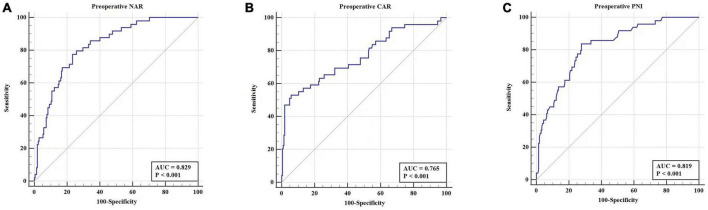
The diagnostic abilities of preoperative values of NAR **(A)**, CAR **(B)**, PNI **(C)** in predicting POD among patients following THA.

The diagnostic abilities of Alb-associated nutritional markers (NAR, CAR, and PNI) were analyzed (Table 3). ROC curve analyses showed that NAR, CAR, and PNI could predict the occurrence of POD with AUC values of 0.829 (cut-off value = 0.12, sensitivity = 77.55%, specificity = 76.59%, and Youden index = 0.5414), 0.765 (cut-off value = 0.19, sensitivity = 53.06%, specificity = 94.15%, and Youden index = 0.4721), and 0.819 (cut-off value = 43.85, sensitivity = 83.67%, specificity = 72.20%, and Youden index = 0.5587), respectively. Totally, the diagnostic abilities of preoperative Alb-associated inflammatory and nutritional markers for POD were good (Figure 2).

**TABLE 3:** Receiver operating characteristic (ROC) curves of preoperative markers for predicting the post-operative delirium among elderly patients following total hip arthroplasty.

Variables	Cut-off values	Sensitivity%	Specificity%	AUC (95%)	Youden index
NAR	0.12	77.55	76.59	0.829	0.5414
CAR	0.19	53.06	94.15	0.765	0.4721
PNI	43.85	83.67	72.20	0.819	0.5587

AUC, area under curve; NAR, neutrophil/albumin ratio; CAR, CRP/albumin ratio, PNI, prognostic nutritional index.

### 3.3. Predictive factors of POD

**FIGURE 3:**
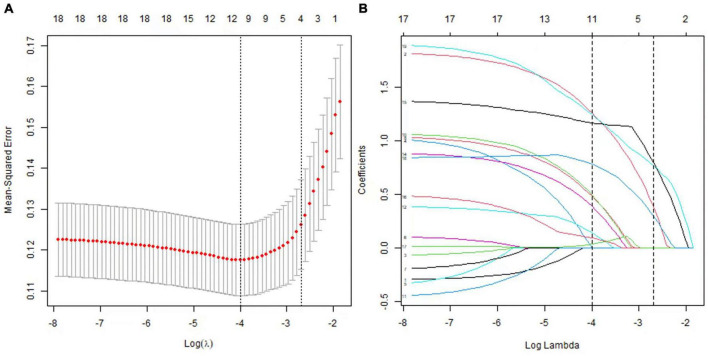
Predictor selection using the LASSO logistic regression model. **(A)** Identification of the optimal penalization coefficient lambda in the Lasso model using 10-fold cross-validation and the minimum criterion. **(B)** Lasso coefficient profiles of the potential predictors.

**FIGURE 4:**
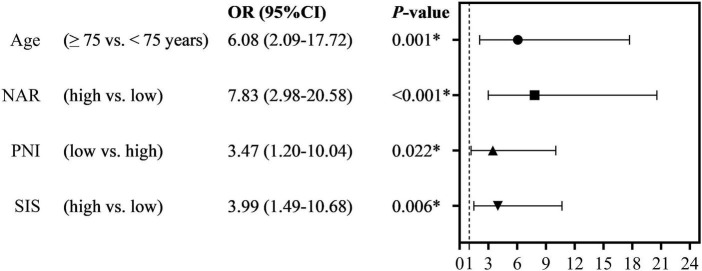
The multivariate logistic regression analyses of the independent predictor of POD (*indicated that they showed statistical differences).

LASSO regression, univariate and multivariate logistic analyses were to obtain risk factors of POD. Univariate logistic analysis indicated that age >75 years, high NAR, high neutrophil, low PNI, low Alb, and high SIS were risk factors for POD (Supplementary Table 3). LASSO regression (Figure 3) indicated that age, NAR, PNI, and SIS were selected into multivariate logistic analysis. In addition, the tolerance was >0.1, and VIF was <5 for the abovementioned factors (Supplementary Table 4), suggesting no collinearity among these variables. Multivariate logistic analyses (Figure 4) showed that age >75 years, high NAR, low PNI, and high SIS could predict POD in elderly patients following THA (Table 4).

**TABLE 4:** Multivariate logistics regression analyses for post-operative delirium in elderly patients following total hip arthroplasty.

Variables	B	S.E.	Wals	OR (95% CI)	*P*-value
Age (≥75 vs. <75 years)	1.805	0.546	10.928	6.08 (2.09–17.72)	0.001*
NAR (high vs. low)	2.058	0.493	17.408	7.83 (2.98–20.58)	<0.001*
PNI (low vs. high)	1.245	0.542	5.275	3.47 (1.20–10.04)	0.022*
SIS (high vs. low)	1.385	0.502	7.612	3.99 (1.49–10.68)	0.006*

*P-value < 0.05. NAR, neutrophil/albumin ratio; PNI, prognostic nutritional index; SIS, systemic inflammation score.

**FIGURE 5:**
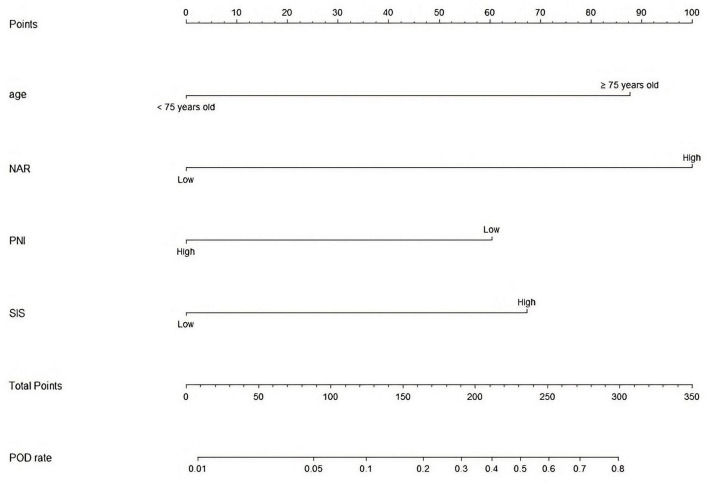
A predictive nomogram model for the prediction of POD.

**FIGURE 6:**
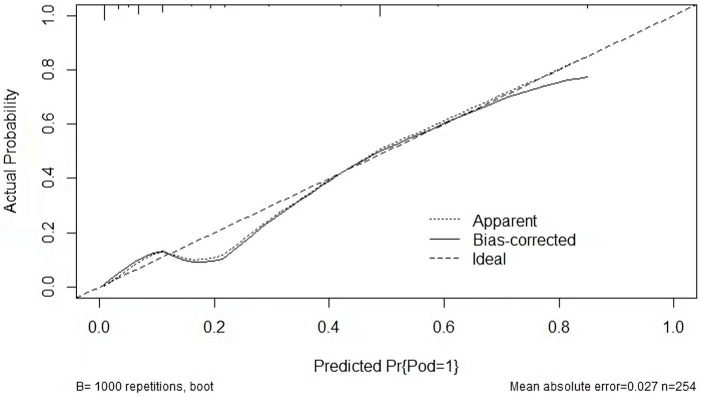
Calibration curve of the prediction nomogram.

**FIGURE 7:**
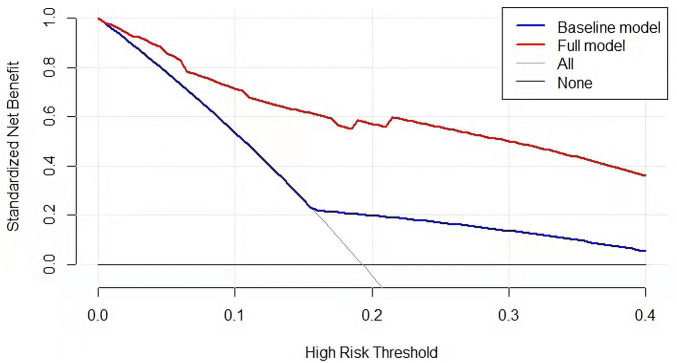
Decision curve for nomogram model to predict the risk of POD.

Last, we developed a nomogram model to predict POD in older patients following THA. Factors from LASSO regression and multivariate logistic analyses were utilized to develop it. The nomogram model was based on age (≥75 vs. <75 years), NAR (high vs. low), PNI (low vs. high), and SIS (high vs. low) (Figure 5). This model showed a good discriminatory ability with a c-index value of 0.869 (95% CI = 0.820–0.918). The calibration curve showed that the nomogram predictive model indicated a good consistency between the observational probability and predicted probability (Figure 6). The DCA suggested that this model had net benefits for the prediction of POD in elderly patients following THA (Figure 7).

## 4. Discussion

This study showed that age >75 years, and Alb-associated inflammatory and nutritional markers (NAR, PNI, and SIS) were predictive factors for POD in elderly patients following THA, suggesting that evaluating the preoperative inflammatory and nutritional status was necessary for elderly patients following THA.

### 4.1. Alb

Previous studies have demonstrated that hypoalbuminemia was significantly associated with an increased risk of delirium (18, 20–22). Chu et al. showed that Alb ≤ 32.26 g/L was a predictive factor for POD in cases with hip fracture (23). Another study from India suggested that Alb ≤ 35 g/L could predict POD in geriatric patients with hip fracture (24). In this study, we observed that Alb ≤ 39.8 g/L could not predict the POD among the elderly patients following THA. Obviously, the Alb level in this study was remarkedly higher than that of other studies, which may be a potential reason for inconsistent results. It is of note that PNI, CAR, SIS, and NAR consisting of Alb may be more accurate markers that reflected the physical condition including inflammation and nutrition status in predicting post-operative complications than a single index such as Alb (25). Therefore, we investigated aforementioned biomarkers including PNI, CAR, SIS, and NAR in this study. The associations between these indicators and POD are summarized in Supplementary Table 2.

### 4.2. PNI

Malnutrition was a risk factor for POD after primary total joint arthroplasty (24, 26–28). PNI, a new nutritional indicator, was calculated by lymphocyte count and serum Alb level. The impact of PNI on post-operative complications including POD following orthopedic surgery was reported before (29, 30). Acarbaş et al. found that preoperative PNI was associated with perioperative adverse events including POD in patients undergoing spinal surgery (29), which was replicated in their further study (30). Several Japanese studies observed an association between PNI and POD after orthopedic surgery (31–34); however, a study by Kobayashi et al. did not (35). We thought different sample sizes, clinical heterogeneity, different definitions of POD may explain these inconsistencies. Chen et al. from China found that preoperative PNI was related to POD after total joint arthroplasty (36). Xing et al. showed that preoperative PNI was related to POD after hip fracture surgery (37). In this study, we showed that low preoperative PNI was an independent predictor for POD following THA. In addition, preoperative PNI could predict POD of non-cardiac surgery (38) and colorectal cancer surgery (39).

### 4.3. CAR

Regarding CAR, Yang et al. indicated that preoperative CAR was not related to the occurrence of POD in cases undergoing lumbar spinal surgery (40). Peng et al. found that preoperative CAR was a significant predictor for POD for older patients receiving total joint arthroplasty (41). A Korean study indicated that preoperative CAR had an association with POD in older patients undergoing hip fracture surgery (42). In addition, Zhang et al. suggested that preoperative CAR may predict POD in older patients (>60 years old) after total knee arthroplasty (43). However, we thought the findings by Zhang et al. were controversial (43). In multivariate logistic regression analyses, they showed that the *P*-value for CAR was larger than 0.05 (43), indicating that CAR was not an independent risk factor for POD. Herein, we showed that patients with a high CAR had a higher percentage of POD after THA in this study. However, the lasso regression analysis showed that preoperative CAR could not predict POD for older patients receiving THA in this study. Further studies in other races are urgently needed in the future.

### 4.4. NAR

NAR, consisting of neutrophil and Alb, is a promising biomarker for predicting the prognosis of cancer (44–47). Günay et al. showed that higher NAR after lower extremity amputation was significantly associated with early mortality after extremity amputation (48). Chen et al. found that NAR was related to all-cause mortality in patients with stroke (49). A study revealed that NAR was associated with a higher risk of low cognitive performance (50). In addition, NAR could reflect the increased inflammatory status in patients with schizophrenia (51). Xie et al. showed that NAR was a reliable biomarker for post-operative complications in patients with colorectal cancer after surgical treatment (52). Up to date, no studies have explored the relationship between NAR and POD in older cases after THA. In this study, we found that high CAR could predict POD for older patients receiving THA. NAR could reflect the status of inflammation and nutrition; thus, higher NAR may indicate the higher inflammation and malnutrition of patients. It may be the reason why higher NAR was significantly associated with POD in patients with THA.

### 4.5. SIS

SIS consisted of Alb and LMR, which could also reflect nutrition and inflammation status. SIS was mainly reported to be associated with the survival of cancers (53–55). In addition, SIS was associated with post-operative complications in surgical patients (56–58). Therefore, we intended to investigate the association between SIS and POD among elderly patients receiving THA. Results of this study showed that high SIS was significantly related with the risk of POD after THA among older patients. To the knowledge, this is the first study to show that preoperative SIS could predict POD in older patients after THA. Further studies in other races and regions should be conducted in future.

### 4.6. Other factors

Inflammation may play a dominant role in the development of delirium. In a recent meta-analysis by Wang et al., they showed that CRP showed a significant association with POD (59). Adamis et al. indicated that CRP was a significant predictor for the types of surgery; however, this effect was predominant in the acute orthopedic surgery and elective abdominal surgery (60). In this study, we found that preoperative CRP could not predict POD among elderly patients after THA. The surgery may aggravate inflammatory response with elevated levels of CRP. Therefore, we thought that the association between post-operative CRP and POD may become stronger. However, we did not evaluate post-operative CRP in this study. Last but not least, this study showed that older age (≥75 years) was a significant predictor for POD after THA. Advanced age was regarded as an accepted predictor for delirium (3, 61). However, Zhang et al. suggested that older age was not a risk factor for POD after total knee arthroplasty (43).

Last, we performed a nomogram predictive model to assess the POD based on the multivariate logistic regression analyses. The C-index value of this model was 0.869 (95% CI = 0.820–0.918). The calibration curve showed that the nomogram model indicated a good consistency between the observational probability and predicted probability. The DCA suggested that this model had net benefits for the prediction of POD in elderly patients following THA.

This study showed several limitations. First, our study was retrospective; thus, a potential selection bias may exist. Second, the sample size was not large enough, which may underpower the findings in this study. Third, post-operative factors that affecting the development of POD were not investigated in this study. Last, we did not split the dataset into training and validation datasets during the modeling process due to the small sample size in this study. The lack of external validation for the predictive model was a limitation of this study.

## 5. Conclusion

This study observes an association between Alb-associated inflammatory and nutritional markers (NAR, PNI, and SIS) and the occurrence of POD in elderly patients following THA. Prospective studies are needed to explore preoperative inflammatory and nutritional markers of POD.

## Data availability statement

The raw data supporting the conclusions of this article will be made available by the authors, without undue reservation.

## Ethics statement

The studies involving humans were approved by the Ethics Committees of the Affiliated Huaian No. 1 People’s Hospital of Nanjing Medical University. The studies were conducted in accordance with the local legislation and institutional requirements.

## Author contributions

YH and JW: study design and project administration. WH: data collection and analysis, and writing. ZS, HS, and JW: revised the manuscript. All authors approved the final manuscript.
