# Supplementary figures and images for: Inflammatory and nutritional markers predict the risk of post-operative delirium in elderly patients following total hip arthroplasty
Source: Front Nutr. 2023 Nov 2;10:1158851. doi: 10.3389/fnut.2023.1158851 (PMC10651730; doi:10.3389/fnut.2023.1158851)

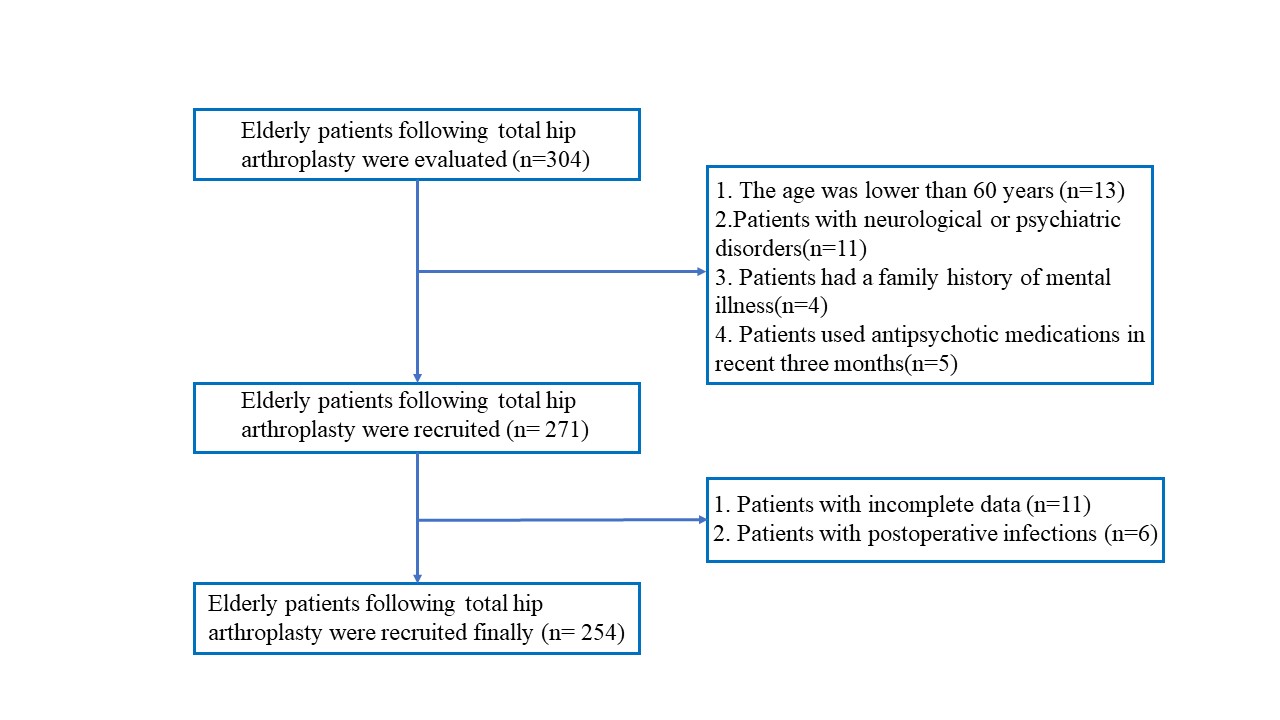

Supplement: Supplementary file 1 [file Image_1.jpg]
