# Supplementary material for: Inflammatory and nutritional markers predict the risk of post-operative delirium in elderly patients following total hip arthroplasty
Source: Front Nutr. 2023 Nov 2;10:1158851. doi: 10.3389/fnut.2023.1158851 (PMC10651730; doi:10.3389/fnut.2023.1158851)
Supplement: Supplementary file 2 [file Table_1.docx]

**Supplementary Table 1.** The definitions of NLR, LMR, NAR, SIS, CAR, and PNI.

| **Variables** | **Definitions** |
| --- | --- |
| NLR | Neutrophil/lymphocyte ratio |
| LMR | Lymphocyte/monocyte ratio |
| NAR | Neutrophil/albumin ratio |
| CAR | CRP/albumin ratio |
| PNI | Albumin(g/L) + 5 * total lymphocyte counts (10^9^/L) |
| SIS=0 | LMR ≥ 2.17 and albumin ≥ 39.8 g/L |
| SIS=1 | LMR < 2.17 or albumin < 39.8 g/L |
| SIS=2 | LMR < 2.17 and albumin < 39.8 g/L |

CRP, C-reactive protein; NLR, neutrophil/lymphocyte ratio; LMR, lymphocyte/ monocyte ratio; NAR, Neutrophil/albumin ratio; CAR, CRP/Albumin ratio; PNI, prognostic nutritional index; SIS, systemic inflammation score
