# Supplementary material for: Inflammatory and nutritional markers predict the risk of post-operative delirium in elderly patients following total hip arthroplasty
Source: Front Nutr. 2023 Nov 2;10:1158851. doi: 10.3389/fnut.2023.1158851 (PMC10651730; doi:10.3389/fnut.2023.1158851)
Supplement: Supplementary file 3 [file Table_2.docx]

**Supplementary Table 2** The association between PNI, CAR, and NAR and the risk of POD in eligible studies.

| **Items** | **Author and year** | **Association with the risk of POD** |
| --- | --- | --- |
| PNI | Acarbas et al, 2019^29^ | Yes* |
|  | Acarbas et al, 2021^30^ | Yes* |
|  | Kurosu et al, 2021^31^ | Yes |
|  | Oe et al, 2019^32^ | Yes |
|  | Oe et al, 2020^33^ | Yes |
|  | Onuma et al, 2020^34^ | Yes |
|  | Kobayashi et al, 2020^35^ | No |
|  | Chen et al, 2021^36^ | Yes |
|  | Xing et al, 2020^37^ | Yes |
|  | Liu et al, 2021^38^ | Yes |
|  | Tei et al, 2010^39^ | Yes |
| CAR | Yang et al, 2022^40^ | No |
|  | Peng et al, 2019^41^ | Yes |
|  | Kim et al, 2023^42^ | Yes |
|  | Zhang et al, 2022^43^ | Yes |
| NAR | Xie et al, 2022^52^ | Yes# |

NAR, neutrophil/albumin ratio; CAR, CRP/albumin ratio; PNI, Prognostic Nutritional Index

*These studies showed an association between PNI and postoperative complications including POD, but not specific for POD

# The study by Xie et al. showed an association between NAR and postoperative complications, but not clear about whether they included POD
