# Supplementary material for: Inflammatory and nutritional markers predict the risk of post-operative delirium in elderly patients following total hip arthroplasty
Source: Front Nutr. 2023 Nov 2;10:1158851. doi: 10.3389/fnut.2023.1158851 (PMC10651730; doi:10.3389/fnut.2023.1158851)
Supplement: Supplementary file 4 [file Table_3.docx]

**Supplementary Table 3.** Univariate logistics regression analyses for postoperative delirium in elderly patients following total hip arthroplasty.

| **Variables** | **B** | **S.E.** | **Wals** | **OR (95%CI)** | ***P* value** |
| --- | --- | --- | --- | --- | --- |
| Gender (Male vs. Female) | -0.324 | 0.348 | 0.865 | 0.72 (0.37-1.43) | 0.352 |
| Age (≥ 75 vs. < 75 years) | 1.487 | 0.405 | 13.470 | 4.42 (2.00-7.79) | <0.001* |
| BMI ((≤ 24 vs. > 24) | -0.366 | 0.319 | 1.314 | 0.69 (0.37-1.30) | 0.252 |
| Smoking (Yes vs. No) | 0.272 | 0.596 | 0.209 | 1.31 (0.41-4.22) | 0.648 |
| Drinking (Yes vs. No) | 0.790 | 1.066 | 0.549 | 0.45 (0.56-3.67) | 0.459 |
| Hypertension (Yes vs. No) | 0.043 | 0.340 | 0.016 | 1.04 (0.54-2.03) | 0.898 |
| Diabetes mellitus (Yes vs. No) | -0.040 | 0.484 | 0.007 | 0.96 (0.37-2.48) | 0.934 |
| Surgery duration (≥ 1.78 vs. < 1.78 hours) | 0.545 | 0.325 | 2.811 | 1.72 (0.91-3.26) | 0.094 |
| Anesthesia duration (≥ 2.11 vs. < 2.11 hours) | 0.459 | 0.323 | 2.029 | 1.58 (0.84-2.98) | 0.154 |
| Neutrophil (high vs. low) | 1.646 | 0.382 | 18.610 | 5.19 (2.46-10.95) | <0.001* |
| Monocyte (high vs. low) | 0.401 | 0.322 | 1.545 | 1.49 (0.79-2.81) | 0.214 |
| Lymphocyte (low vs. high) | 0.779 | 0.331 | 5.534 | 2.18 (1.14-4.17) | 0.019 |
| Albumin (low vs. high) | 2.157 | 0.432 | 24.896 | 8.64 (3.70-20.16) | <0.001* |
| CRP (high vs. low) | -0.362 | 0.322 | 1.259 | 0.70 (0.37-1.31) | 0.262 |
| NAR (high vs. low) | 2.334 | 0.458 | 25.939 | 10.32 (4.20-25.35) | <0.001* |
| LMR (low vs. high) | 1.004 | 0.340 | 8.711 | 2.73 (1.40-5.32) | 0.003 |
| CAR (high vs. low) | 0.132 | 0.319 | 0.173 | 1.13 (0.61-2.13) | 0.678 |
| PNI (low vs. high) | 2.137 | 0.432 | 24.443 | 8.47 (3.63-19.76) | <0.001* |
| SIS (high vs. low) | 2.236 | 0.398 | 31.566 | 9.36 (4.29-20.42) | <0.001* |

* *P* value < 0.05

NAR, neutrophil/albumin ratio; PNI, Prognostic Nutritional Index; SIS, Systemic Inflammation Score.
