# Supplementary material for: Inflammatory and nutritional markers predict the risk of post-operative delirium in elderly patients following total hip arthroplasty
Source: Front Nutr. 2023 Nov 2;10:1158851. doi: 10.3389/fnut.2023.1158851 (PMC10651730; doi:10.3389/fnut.2023.1158851)
Supplement: Supplementary file 5 [file Table_4.docx]

**Supplementary Table 4** The tolerance and VIF for age, NAR, PNI, and SIS.

| **Items** | **tolerance** | **VIF** |
| --- | --- | --- |
| Age | 0.992 | 1.008 |
| NAR | 0.931 | 1.075 |
| PNI | 0.579 | 1.727 |
| SIS | 0.589 | 1.699 |

VIF, variance inflation factor; NAR, neutrophil/albumin ratio; PNI, Prognostic Nutritional Index; SIS, Systemic Inflammation Score.
